# Molecular characterization of penicillin non-susceptible *Streptococcus pneumoniae* isolated before and after pneumococcal conjugate vaccine implementation in Casablanca, Morocco

**DOI:** 10.1186/s12941-017-0200-6

**Published:** 2017-04-04

**Authors:** Idrissa Diawara, Abouddihaj Barguigua, Khalid Katfy, Kaotar Nayme, Houria Belabbes, Mohammed Timinouni, Khalid Zerouali, Naima Elmdaghri

**Affiliations:** 1 Laboratoire de Microbiologie, Faculté de Médecine et de Pharmacie, Hassan II University of Casablanca, B.P 5696, Casablanca, Morocco; 2 Service de Microbiologie, CHU Ibn Rochd, B.P 2698, Casablanca, Morocco; 3 Laboratoire Polyvalent en Recherche et Développement, département de Biologie-Géologie, Faculté polydisciplinaire, Université Sultan Moulay Slimane, Beni Mellal, Morocco; 4 Molecular Bacteriology Laboratory, Institut Pasteur du Maroc, Casablanca, Morocco

**Keywords:** *Streptococcus pneumoniae*, Invasive pneumococcal disease, Penicillin-binding proteins, β-lactams, Serotypes, Antibiotic resistance, PFGE

## Abstract

**Background:**

*Streptococcus pneumoniae* is a major cause of morbidity and mortality worldwide, especially among children and the elderly. The ability to effectively treat pneumococcal infection has been compromised due to the acquisition of antibiotic resistance, particularly to β-lactam drugs. This study aimed to describe the prevalence and molecular evolution of penicillin non-susceptible *S. pneumoniae* (PNSP) isolated from invasive diseases before and after pneumococcal conjugate vaccine implementation in Casablanca, Morocco.

**Methods:**

Isolates were obtained from the Microbiology Laboratory of Ibn Rochd University Hospital Centre of Casablanca. Serogrouping was done by Pneumotest Kit and serotyping by the Quellung capsular swelling. Antibiotic susceptibility pattern was determined by disk diffusion and E-test methods. The PNSP were analyzed by pulsed-field gel electrophoresis (PFGE) and by genotyping of *pbp1a*, *pbp2b*, and *pbp2x* genes.

**Results:**

A total of 361 *S. pneumoniae* isolates were collected from 2007 to 2014. Of these isolates, 58.7% were obtained before vaccination (2007–2010) and 41.3% after vaccination (2011–2014). Of the 361 isolates, 80 were PNSP (22.2%). Generally, the proportion of PNSP between pre- and post-vaccination periods were 31 and 13% (*p* = 0.009), respectively. The proportion of PNSP isolated from pediatric and adult (age > 14 years) patients decreased from 34.5 to 22.9% (*p* = 0.1) and from 17.7 to 10.2% (*p* = 0.1) before and after vaccine implementation, respectively. The leading serotypes of PNSP were 14 (33 vs. 57%) and 19A (18 vs. 14%) before and after vaccination among children. For adults, serotypes 19A (53%) and 23F (24%) were the dominant serotypes in the pre-vaccination period, while serotype 14 (22%) was the most prevalent after vaccination. There were 21 *pbp* genotypes in the pre-vaccination period vs. 12 for post-vaccination period. PFGE clustering showed six clusters of PNSP grouped into three clusters specific to pre-vaccination period (clusters I, II and III), two clusters specific to post-period (clusters V and VI) and a cluster (IV) that contained clones belonging to the two periods of vaccination.

**Conclusion:**

Our observations demonstrate a high degree of genetic diversity among PNSP. Genetic clustering among PNSP strains showed that they spread mainly by a restricted number of PNSP clones with vaccine serotypes. PFGE clustering combined with *pbp* genotyping revealed that vaccination can change the population structure of PNSP.

## Background

*Streptococcus pneumoniae* is a major cause of morbidity and mortality worldwide, especially among children and the elderly. Pneumococcal infections include serious diseases such as meningitis, bacteraemia, and pneumonia, as well as milder but more common illnesses, such as sinusitis and otitis media [1]. Disease rates and mortality are higher in developing than in industrialized settings, with the majority of deaths occurring in Africa and Asia. The ability to effectively treat pneumococcal infection has been compromised due to the acquisition of antibiotic resistance, particularly to β-lactam drugs [2].

Furthermore, antibiotic pressure, in combination with these horizontal recombination events, allows the acquisition of antibiotic-resistant genes or resistant strains which increases the resistance to a variety of antibiotics [3]. Pneumococcal resistance to β-lactams has been attributed to alterations of the penicillin-binding proteins (PBP) which reduce their affinity [4]. The first pneumococcal isolate resistant to penicillin was reported in 1967 from a patient in Australia [5], and resistant pneumococci have subsequently increased in prevalence worldwide. β-Lactam antibiotics exert their biological effects by interacting with the PBPs. PBPs are membrane enzymes that catalyze the polymerization and transpeptidation of glycan strands, during the assembly of the bacterial cell wall. β-Lactam resistance in clinical pneumococci is mediated by altered PBPs, specifically PBP1a, PBP2x and PBP2b [6, 7]. Penicillin resistance in *S. pneumoniae* is mediated by stepwise alterations of PBPs [8–10]. These three PBPs are considered to be the key targets for these agents and were therefore chosen for examination in this study.

In Morocco, the PCV-13 was introduced in the national immunization program in October 2010 in 2 + 1 schedule and replaced by the PCV-10 in July 2012. Before pneumococcal vaccine implementation in Morocco, the incidence rate of invasive pneumococcal diseases (IPD) in children aged to 2 years was 34.6/100,000 populations. The incidence rates of PCV-7, PCV-10 non-PCV-7 and PCV-13 non-PCV-10 serotypes were 18.0, 5.7 and 5.7/100,000 population in the same age, respectively [11]. In 2010, the use of the pneumococcal conjugate vaccine (PCV-13 and then PCV-10) dramatically reduced the prevalence of vaccine serotypes through active vaccination particularly among children less than 5 years of ages in Casablanca, Morocco. However, the introduction of vaccination was associated with a subsequent relative increase in non-vaccine serotypes [11]. This can be attributed to the phenomena of “serotype replacement”, the expansion of preexisting NVT pneumococci, and/or serotype switching [12]. Vaccination has also reduced the incidence rate of antibiotic resistant serotypes, but we previously reported, a rebound due to the persistence of some vaccine serotypes like 6B and 14 [11]. Although several studies have described the genetic profile of the *pbp1a*, *pbp2b* and *pbp2x* genes in pneumococci from different countries [4, 13], actually, there are no studies on the characteristics of penicillin non- susceptible *S. pneumoniae* (PNSP) in Morocco.

This study aimed to describe the molecular evolution of penicillin non-susceptible *S. pneumoniae* isolated from invasive diseases before and after pneumococcal conjugate vaccine implementation in Morocco in 2010.

## Methods

### Bacterial strains, growth conditions and DNA extraction

Isolates, collected from 2007 to 2014, were obtained from the Microbiology Laboratory of Ibn Rochd University Hospital Centre of Casablanca (IR-UHC). All the non-duplicate invasive *S. pneumoniae* isolates recovered from patients, all ages included, during the study periods were included. Isolates obtained from normally sterile sites [cerebrospinal fluid (CSF), blood, pleural fluids, articular fluids or any other sterile site] were considered invasive.

The isolates were identified based on the typical colony morphology, Gram staining, optochin sensitivity test (Oxoid Company, Britain) on Mueller–Hinton agar plates supplemented with 5% sheep blood (BioMèrieux, Lyon, France) and bile solubility. The procedures employed for capsular typing and DNA extraction were previously described [14].

### Antimicrobial susceptibility

Antibiotic susceptibility testing was done following Clinical Laboratory Standard Institute guidelines (CLSI, 2014). Erythromycin, tetracycline, chloramphenicol, and trimethoprim-sulfamethoxazole (cotrimoxazole), were tested by disk diffusion with antibiotic disks from Oxoid (Basingstoke, United Kingdom) on Mueller-Hinton Agar supplemented with 5% sheep blood (BioMèrieux, Lyon, France). A minimal inhibitory concentration (MIC) for penicillin G and ceftriaxone was determined on 5% sheep blood Mueller-Hinton agar with E-tests from Oxoid (Oxoid, Basingstoke, UK). The breakpoints used for interpretation were those recommended by the CLSI in 2014. Quality control was conducted using *S. pneumoniae* ATCC 49619.

### PCR- RFLP of *pbp* genes

Genetic polymorphism of the penicillin resistance genes *pbp1a*, *pbp2b*, and *pbp2x* of the penicillin-nonsusceptible isolates was investigated by restriction fragment length polymorphism (RFLP) analysis as described previously [15]. Briefly, we amplified a segment of 2.4, 1.5, and 2 kb of *pbp1a, pbp2b*, and *pbp2x* genes respectively by PCR. PCR amplifications were performed in simplex in a 25 μL reaction mixture containing 0.5 mM of dNTPs, 0.3 μM of each primer, 1× of PCR buffer, 2.5 mM of MgCl_2_, 1U of Platinum *Taq* DNA polymerase (Invitrogen). The PCR cycle was 95 °C for 4 min followed by 30 amplification cycles of 94 °C for 1 min, 58 °C (*pbp1a*), 55 °C (*pbp2b*) and 60 °C (*pbp2x*) for 2 min, and 72 °C for 3 min; the final extension was 72 °C for 10 min. The amplification products were digested by restriction endonuclease *Hae*III and *Rsa*I and separated by agarose gel electrophoresis. Gels were scanned and analyzed by the Geldoc system (Bio-Rad). The different *pbp* genotypes received a three numbers code (e.g., x/y/z) referring to the RFLP patterns of the genes *pbp1a* (x*), pbp2b* (y), and *pbp2x* (z), respectively. As positive control for the three genes, we used 15 penicillin-susceptible *S. pneumoniae* (PSSP).

### Pfge

PFGE was performed to determine the genetic relatedness among the same *pbp* genotypes of pneumococcus strains isolated before and after vaccination, following a standardized protocol developed by Bean et al. [16], Elliot et al. [17] and according to the recommendations of the pneumococcal molecular epidemiology network (PMEN). The gel images were processed and analyzed by BioNumerics Ver. 7.5 software (Applied Maths, Belgium). The images were normalized by use of standard molecular markers, and banding patterns were compared. Similarity analysis was performed using Dice coefficients and isolates were separated into similarity clusters by the unweighted-pair group method using average linkages (UPGMA).

### Statistical analysis

Data were analyzed with WHONET5.6, EpiInfo 7 (Centers for Disease Control, Atlanta, Georgia, USA) and Microsoft Excel. The Chi square test or Fisher’s exact test was performed to compare proportion between collection periods. Differences were considered significant if the *p* value was <0.05.

## Results

### Prevalence of PNSP

A total of 361 *S. pneumoniae* isolates were collected from 2007 to 2014. Of these isolates, 58.7% were obtained before vaccination (2007–2010) and 41.3% after vaccination (2011–2014). Considering before and after vaccine introduction periods, isolates recovered from children (aged from 0 to 14 years) represented 54.7 and 41%, respectively. Of the 361 isolates, 80 were PNSP (22.2%). Consecutive PNSP, one per patient, were collected from blood cultures (43.5%), cerebrospinal fluid (40%), pleural fluid (4%) and other sterile body fluids (12.5%). Generally, the proportion of PNSP between pre- and post-vaccination periods were 31% and 13% (*p* = 0.009), respectively. The proportion of PNSP isolated from pediatric and adult (age ≤ 14 years) patients decreased from 34.5 to 22.9% (*p* = 0.1) and from 17.7% to 10.2% (*p* = 0.1) before and after vaccine implementation, respectively.

Of the 80 PNSP, according to CLSI breakpoints, we found co-resistance to other antibiotics: before vaccination, the proportion of PNSP resistant to cotrimoxazole was 66.7%, 40% to tetracycline, 21% to erythromycin and 5.1% to chloramphenicol and ceftriaxone. As for the post-vaccination period, among the 23 PNSP, 56.5% were resistant to cotrimoxazole, 47.8% to erythomycin and tetracycline, and 8.7% to ceftriaxone (intermediate susceptibility).

### Serotype distribution among PNSP strains

**Fig. 1:**
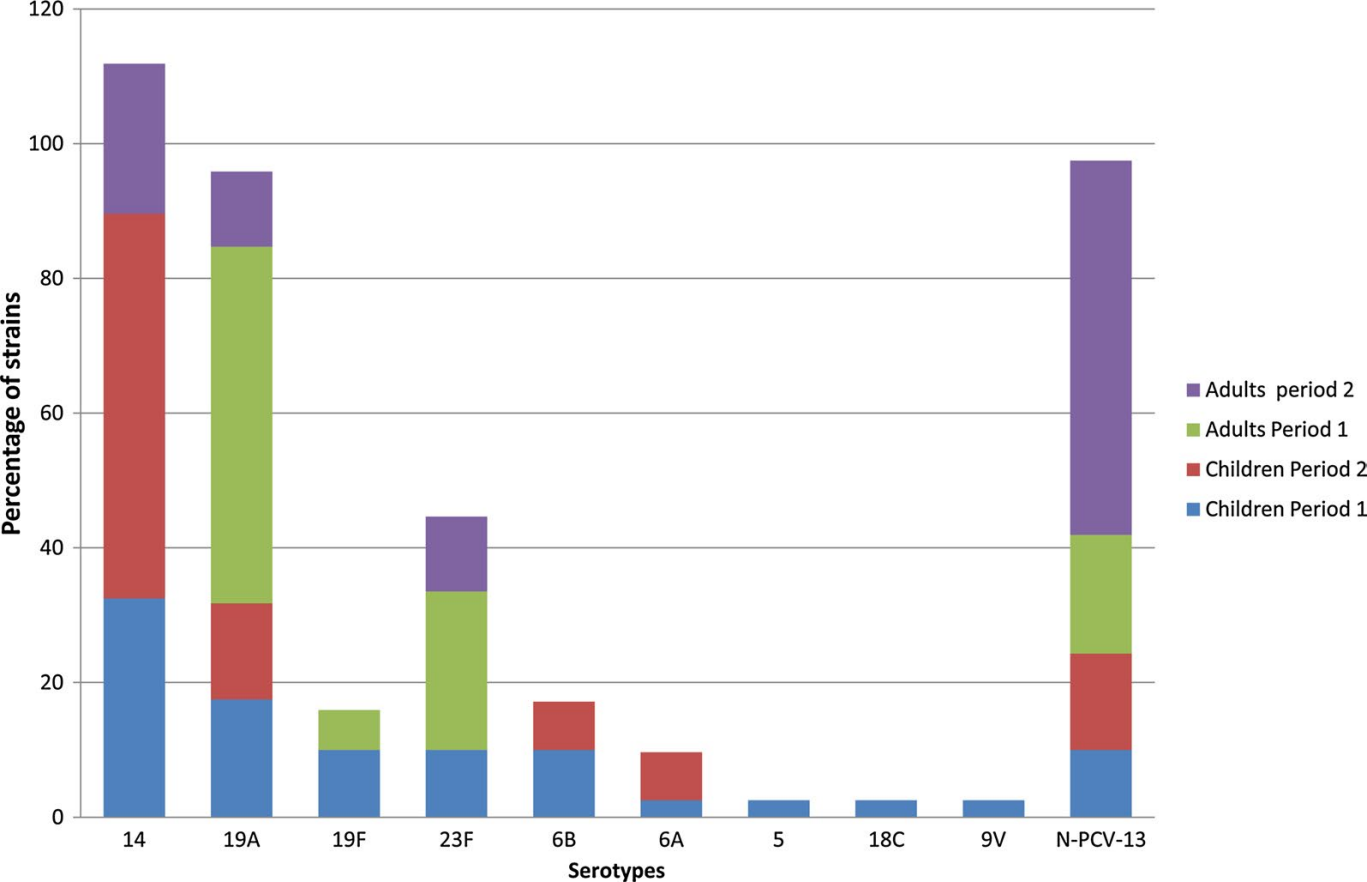
Serotype distribution of the 80 penicillin non-susceptible *S. pneumoniae* isolated from IPD among children and adult, before and after vaccination in Casablanca, Morocco. Period 1 is the pre-vaccination period from 2007 to 2010 and Period 2 is the post-vaccination period from 2011 to 2014. The total number of PNSP was 40 and 14 strains for children, and 17 and 9 strains for adult, for period 1 and period 2, respectively

Serotype distribution showed that vaccine serotypes and non-vaccine serotypes represented 90 and 10% among the PNSP isolated in children before vaccination; after, they represented 85.7 and 14.3%, respectively. In the adult population, vaccine and non-vaccine serotypes accounted for 82.4 and 17.6% before vaccination, while they represented 44.4 and 55.6% of PNSP after vaccination, respectively. The leading serotypes were 14 (33 vs. 57%) and 19A (18 vs. 14%) before and after vaccination among children. For adults, serotypes 19A (53%) and 23F (24%) were the dominant serotypes in the pre-vaccination period, while serotype 14 (22%) was the most prevalent after vaccination (Fig. 1).

### *pbp* genotypes

**Fig. 2:**
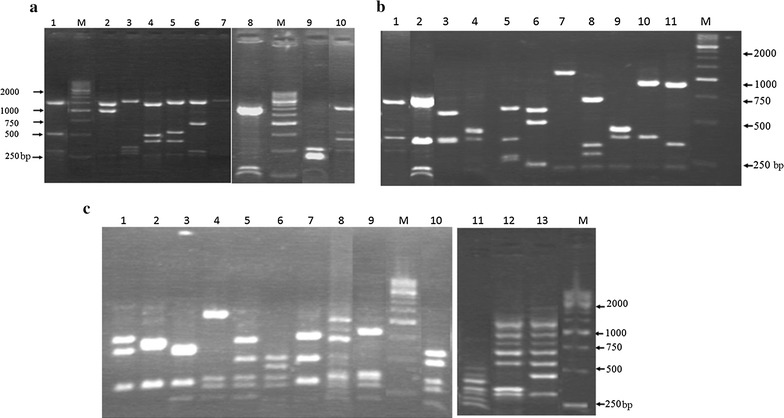
PCR-RFLP profiles of the different *pbp* genes digested by *Hae*III and *Rsa*I. **a** Profile of *pbp1a* gene, *lanes* 1–10 correspond to RFLP profiles; **b** profile of *pbp2b* gene, *lanes* 1–11 correspond to RFLP profiles. **c** Profile of *pbp2x* gene, *lanes* 1–13 correspond to RFLP profiles. *Line M* corresponds to DNA size standards (250 base pairs)

A total of 10, 11 and 13 restriction profiles were found among the 80 PNSP strains after analysis of *pbp* gene by PCR–RFLP specific to *pbp1a*, *pbp2b*, and *pbp2x*, respectively (Fig. 2). For the 15 PSSP, control strains, we found only one profile for *pbp1a* while *pbp2b* and *pbp2x* presented different profiles with 4 and 3 profiles respectively (data none shown). *pbp* genotype of each strain was determined by combining the profiles of the three genes. This combination allowed to classify the strains collected in the pre-vaccination period into 21 different genotypes versus 12 for post-vaccination period. The different genotypes found during the two periods were illustrated in the Table 1. In general, the diversity of *pbp* genotype was associated to serotypes: serotype-dependent. This was highlighted for *pbp* genotype 1/5/1 associated to serotype 14 and for *pbp* genotype 4/2/5 associated to serotype 19A. However, several serotypes, grouped in a single genotype, were found in the two periods of vaccination.

**Table 1:** PBP genotypes and serotypes distribution of the 80 penicillin non-susceptible *Streptococcus pneumoniae* isolated from invasive diseases in Casablanca, Morocco (2007–2014)

Genotype of pbp genes			Number of strains	MIC for PG (mg/L)	Serotype (number of strains)
*pbp1a*	*pbp2b*	*pbp2x*			
*Pre-vaccination period*					
1	3	7	1	1	23F (1)
*1*	*5*	*7*	*6*	*0.25–2*	*23F (2), 14 (2), 23A (1), 5 (1)*
*1*	*5*	*1*	*3*	*1–2*	*14 (3)*
1	7	7	1	2	9V (1)
1	9	6	1	2	14 (1)
2	4	6	1	2	14 (1)
2	5	7	4	0.5*–*2	14 (4)
*2*	*5*	*12*	*1*	*0.5*	*14 (1)*
2	5	8	1	2	19F (1)
2	5	1	1	2	14 (1)
2	7	8	2	0.5*–*2	6B (2)
*2*	*8*	*1*	*2*	*0.5–2*	*19F (2)*
3	6	9	1	0.25	23F (1)
*4*	*2*	*5*	*17*	*0.125–0.5*	*19A (16), 19F (1)*
*4*	*2*	*2*	*4*	*0.5–1*	*24 (1) NT(2) 19F (1)*
4	7	7	3	0.5	NT (3)
5	6	11	1	0.125−0.5	23F (1)
*6*	*3*	*3*	*2*	*0.125−0.25*	*6B (2)*
6	10	4	3	0.25	NT (3)
8	9	10	1	0.25	6A (1)
10	11	11	1	0.25	18C (1)
*Post-vaccination period*					
1	1	1	1	2	14 (1)
*1*	*5*	*1*	*1*	*2*	*14 (1)*
*2*	*8*	*1*	*1*	*0.5*	*19F (1)*
*4*	*2*	*2*	*6*	*0.25−0.5*	*NT (3), 7F (1), 7A (2)*
*6*	*3*	*3*	*2*	*0.125−1*	*6B (1), 6A (1)*
1	4	6	*1*	2	14 (1)
*4*	*2*	*5*	*2*	*0.25*	*19A (2)*
1	5	6	*1*	2	14 (1)
*1*	*5*	*7*	*5*	*0.5–1*	*14 (5)*
5	2	11	*1*	0.25	23F (1)
*2*	*5*	*12*	*1*	*2*	*14 (1)*
7	6	13	1	0.125	22F (1)

*MIC* minimal inhibitory concentration, *PG* penicillin G, *pbp* penicillin binding protein gene

Genotypes written in italics are those selected for PFGE typing

A total of 58.3% (7/12) of *pbp* genotypes for the post-vaccination period were the same as those found in the pre-vaccination period. The dominant genotype in the pre-vaccination period was 4/2/5, carried by 16 strains of serotype 19A and one serotype19F. As for the post-vaccination period, the prevalent genotype was 4/2/2, carried by 3 strains of non-vaccine serotypes, two strains of serotype 7A and one serotype 7F (Table 1).

### PFGE pulsotypes

**Fig. 3:**
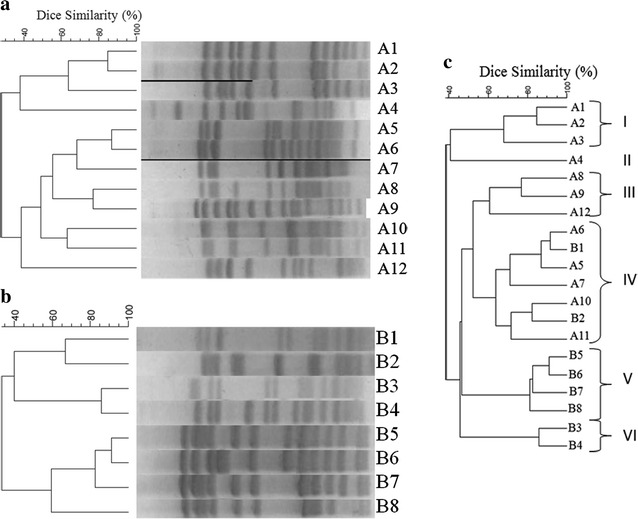
PFGE dendrogram analysis generated by BioNumerics for Molecular typing of penicillin non-susceptible *S. pneumoniae* (PNSP).isolates. **a** PFGE dendrogram for PNSP isolated before vaccination (2007–2010). **b** PFGE dendrogram for PNSP isolated after vaccination (2011–2014). **c** PFGE dendrogram obtained by combined analysis of PNSP pulsotypes found during pre- and post-vaccination periods, the clusters at 60% of dice coefficient are from I to VI

PFGE was performed to determine the genetic relatedness among the same *pbp* genotypes of pneumococcus strains isolated before (n = 12) and after (n = 8) vaccination. Isolates were assigned to the pulsed-field profiles designation as A1 to A12 for the 12 genotypes of the pre-vaccination period and B1 to B8 for the 8 genotypes of the post-vaccination period. The different pulsotypes as well as the associated genotypes are shown in the Table 2. Pulsotypes analysis of the pre-vaccination period, illustrated in Fig. 3a, showed a clonal and polyclonal dissemination of PNSP with 12 different pulsotypes. The pulsotypes A1 and A2 had a Dice similarity greater than 80% therefore belong to the same clone. These two clones belonged to the same serotype (serotype 14) and the same genotype (1/5/1). These clones were isolated only among children. Two other pulsotypes, A5 and A6, with the same serotype (19A) and the same *pbp* genotype (4/2/5), showed clonal dissemination among adults population.

**Table 2:** Pulsotype, PBP genotypes and serotypes distribution of penicillin non-susceptible *Streptococcus pneumoniae* isolated from invasive diseases in Casablanca, Morocco (2007–2014)

Pulsotypes	Genotypes of *pbp* genes			Number of strains	MIC of PG (mg/L)	Serotype (number of strains)
	*plp1a*	*plp2b*	*plp2x*			
*Pre-vaccination period*						
A1, A2	1	5	1	3	1−2	14 (3)
A12	1	5	7	3	0.25−2	23F (2), 23A (1)
ND	1	5	7	1	1	5 (1)
A3	1	5	7	2	1	14 (2)
A4	2	8	1	2	0.5−2	19F (2)
A2	2	5	12	1	0.5	14 (1)
A11	4	2	2	3	0.5−1	24 (1) NT (2)
A9	4	2	2	1	0.5	19F (1)
A6, A5	4	2	5	8	0.125−0.5	19A (8)
A5	4	2	5	3	0.125−0.5	19A (3)
A7	4	2	5	5	0.125−0.5	19A (5)
A8	4	2	5	1	0.125	19F (1)
A10	6	3	3	2	0.125−0.25	6B (2)
*Post-vaccination period*						
B8	1	5	1	2	2	14 (1)
B5, B6, B7	1	5	7	5	0.5−1	14 (5)
B1	2	8	1	1	0.5	19F (1)
B6	2	5	12	1	2	14 (1)
ND	4	2	2	3	0.25−0.5	NT (3)
B8	4	2	2	3	0.25−0.5	7F (1), 7A (2)
B3, B4	4	2	5	2	0.25	19A (2)
B2	6	3	3	2	0.125−1	6B (1), 6A (1)

*MIC* minimal inhibitory concentration, *PG* penicillin G, *pbp* penicillin-binding protein gene, *ND* not determined

A total of 8 pulsotypes were found among PNSP isolated during the post-vaccination period (Fig. 3b). Pulsotypes B3 and B4 constituted a single clone sharing the same *pbp* genotype and the both belonged to serotype 19A. This clone was present in both among adults and children. Pulsotype B5, B6 and B7 constituted a single clonal complex with the same *pbp* genotype and belonging to the serotype 14. This clonal complex diffused only among children.

The combined analysis of PNSP pulsotypes found during pre- and post-vaccination periods showed several clusters, as shown in the Fig. 3c. At the 60% level of Dice similarity, six clusters containing two or more isolates were found. The clusters I, II and III were specific to pre-vaccination period and clusters V and VI were specific to post-vaccination period. Interestingly, between these two cluster groups, there was the cluster IV that contained pulsotypes belonging to the pre- and post-vaccination periods. Analysis of this cluster showed that pulsotypes A6, B1 and A5, although present in the two periods, showed clonal dissemination before and after vaccination. However, while clones A5 and A6 are serotypes 19A, pulsotype B1 is a 19F. The Pulsotypes A10 and B2 also showed clonal dissemination between the two periods. These two clones belonged to serogroup 6 and had the same *pbp* genotypes.

## Discussion

Antibiotic resistance in *S. pneumoniae* is a serious concern globally [18]. One of the added benefits of the PCVs in Casablanca has been the decline in *S. pneumoniae* antibiotic resistance, most notably for the PNSP and cotrimoxazole non-susceptible strains in children under 2 years of age, as reported elsewhere [11]. This is not surprising since the majority of serotypes associated with penicillin resistance in Casablanca are serotypes found in the PCV-10 and PCV-13. Although vaccination has reduced the incidence rate of antibiotic resistant serotypes, but the persistence of some vaccine serotypes constitutes a serious concern and needed to be analyzed.

This study describes the prevalence, serotype distribution, genetic diversity of *pbp* genes and molecular evolution of PNSP isolated in Casablanca before and after PCV-13 and PCV-10 introduction in Moroccan NIP. Vaccine coverage of PCVs vaccines in children aged to 2 years of age was estimated to 88% at the Grand Casablanca in 2014 as declared by the observatory regional of epidemiology service of health of Casablanca. The overall prevalence of PNSP was 22.2% from 2007 to 2014. This rate of resistance decreased from 34.5 to 22.9% and from 17.7 to 10.2% before and after vaccine implementation, among children and adults respectively. Significant reduction of PNSP occurred in several countries after the use of PCV [19–23]. The maximal level of resistance of PNSP responsible of IPD is relatively low with MICs = 2 mg/L compared to MICs of PNSP in many countries where MICs ≥ 8 mg/L were reported [4, 24]. Serotypes among PNSP were mainly 6B, 14, 19A, 19F and 23F in children and adults. These serotypes are included in the currently vaccine program (PCV-10) in Morocco except 19A. As previously published, resistance, particularly high-level penicillin resistance, is mainly associated with seven serotypes commonly carried in children: 6A, 6B, 9V, 14, 19A, 19F, and 23F [25].

Analysis of genetic diversity of *pbp* genes of these serotypes showed a high similarity between pre- and post-vaccine periods. Analyzes of genetic diversity have already been published [15, 26] but this is the first time that a study has analyzed this diversity before and after the introduction of PCV. The genetic profile of the *plp1a*, *plp2b* and *plp2x* genes shows that *plp1a* exhibited high genetic stability followed by *plp2x* and finally *plp2b* as shown by analysis of PSSP strains. However, genetic diversity of *pbp* genes showed that 58.33% of the *pbp* genotypes of post-vaccine period were the same as those found before vaccination. Furthermore, we found that *pbp* genotypes were closely related to serotypes “serotype-dependent”. This close linkage is probably due to the position of the *cps* loci between *pbp1a* gene in upstream and *plp2x* gene in downstream [12, 27]. This configuration could promote co-transfer and a genetic harmony between serotype and *pbp* genotypes.

To study the same *pbp* genotypes found before and after vaccination, we used PFGE. The molecular epidemiology profile, as obtained by PFGE, showed that despite the genotypic diversity of the *pbp* genes in PNSP before vaccination, there was a clonal dissemination. The same situation was observed after vaccination. Thus, prior to vaccination, we found clonal dissemination of serotypes 19A and 14, where serotypes 19A had the same *pbp* genotypes. Depending to the level of penicillin resistance and to the type of *pbp* genotype, the clonal dissemination of serotype 14 was different to serotype 19A. These data suggest that the same PNSP clone can harbor different genotypes of *pbp* genes as previously suggested by Gherardi et al. [15] in the USA. The genotyping of *pbp* genes showed that several serotypes can belong to the same *pbp* genotype. One of the benefits of PFGE method was to differentiate these serotypes in different pulsotypes (clone), which showed a higher degree of resolution of PFGE compared to the genotyping of the *pbp* genes. The prevalence and distribution of serotypes among a population of pneumococci are an important consideration, especially in the context of post-vaccine period to evaluate vaccine efficacy.

In this study, PFGE clustering combined with *pbp* genotyping revealed that vaccination can change the population structure of PNSP. These findings suggest that probably, the clones specific to pre-vaccine periods was eliminated by large scale vaccination in Casablanca. For the clones specific to the post-period, they could be an emerging of new clones that escaped to the vaccination. As for the clones belonging to the clusters IV, they probably escaped to the vaccination and they remained before and persistent after vaccination. Molecular epidemiology study by whole genome sequencing will be the next step of the current study to corroborate our results.

## Conclusions

This study investigated the prevalence of the molecular characteristics of PNSP causing IPD in Casablanca. Our observations demonstrated a high degree of genetic diversity among PNSP. Genetic clustering among PNSP strains showed that they spread mainly by a restricted number of PNSP clones with vaccine serotypes. PFGE clustering combined with *pbp* genotyping revealed that vaccination can change the population structure of PNSP. In Casablanca, as previously published, IPD are reduced by PCVs, this should normally result in a reduction of antibiotic prescription rate in children and adults. Limiting antibiotic prescription and large-scale vaccination using pneumococcal conjugate vaccines containing all vaccine serotypes would probably contribute to control this problem. Surveillance of antibiotic-resistant pneumococci in Casablanca should be continued in the era of multivalent pneumococcal conjugate vaccines, with due attention to the mechanisms of antibiotics resistance.
